# Differential proteomics and physiology of *Pseudomonas putida* KT2440 under filament-inducing conditions

**DOI:** 10.1186/1471-2180-12-282

**Published:** 2012-11-27

**Authors:** Aurélie Crabbé, Baptiste Leroy, Ruddy Wattiez, Abram Aertsen, Natalie Leys, Pierre Cornelis, Rob Van Houdt

**Affiliations:** 1 Unit of Microbiology, Expert Group Molecular and Cellular Biology, Institute for Environment, Health and Safety, Belgian Nuclear Research Centre (SCK CEN), Mol, Belgium; 2 Department of Proteomics and Microbiology, Interdisciplinary Center of Mass Spectrometry (CISMa), University of Mons (UMONS), Mons, Belgium; 3 Laboratory of Food Microbiology and Leuven Food Science and Nutrition Research Centre, Centre for Food and Microbial Technology, Department of Microbial and Molecular Systems, Faculty of Bioscience Engineering, Katholieke Universiteit Leuven, Leuven, Belgium; 4 Laboratory of Microbial Interactions, Department of Molecular and Cellular Interactions, Flanders Institute for Biotechnology (VIB), Vrije Universiteit Brussel, Brussels, Belgium; 5 Present address: The Biodesign Institute, Center for Infectious Diseases and Vaccinology, Arizona State University, 1001 S. McAllister Avenue, Tempe, AZ, 85287, USA

**Keywords:** *Pseudomonas putida* KT2440, Filamentation, Elongation, SOS response, RecA, Shaking speed, Stress resistance

## Abstract

**Background:**

*Pseudomonas putida* exerts a filamentous phenotype in response to environmental stress conditions that are encountered during its natural life cycle. This study assessed whether *P. putida* filamentation could confer survival advantages. Filamentation of *P. putida* was induced through culturing at low shaking speed and was compared to culturing in high shaking speed conditions, after which whole proteomic analysis and stress exposure assays were performed.

**Results:**

*P. putida* grown in filament-inducing conditions showed increased resistance to heat and saline stressors compared to non-filamented cultures. Proteomic analysis showed a significant metabolic change and a pronounced induction of the heat shock protein IbpA and recombinase RecA in filament-inducing conditions. Our data further indicated that the associated heat shock resistance, but not filamentation, was dependent of RecA.

**Conclusions:**

This study provides insights into the altered metabolism of *P. putida* in filament-inducing conditions, and indicates that the formation of filaments could potentially be utilized by *P. putida* as a survival strategy in its hostile, recurrently changing habitat.

## Background

The soil bacterium *Pseudomonas putida* has to cope with diverse and variable habitat-associated stressors to ensure its survival [1]. Besides the exposure of *P. putida* to toxic pollutants and antibacterial compounds in soils, this bacterium encounters osmotic, thermal, oxidative and starvation stresses in the natural habitat [2-5].

Under certain laboratory growth conditions, *P. putida* exerts a filamented phenotype [6]. Filamentation occurs due to the lack of septation during the cell growth process and results in the formation of elongated bacteria, which is typically a consequence of DNA damage or envelope stress [7]. Cell division inhibition is most commonly mediated by the DNA-damage response system (SOS response) [7]. DNA damage (for example, due to ultraviolet irradiation or oxidative radicals) results in the exposure of single-stranded DNA stretches that become covered by the RecA recombinase. In this nucleoprotein filament, RecA becomes activated and stimulates the autoproteolysis of the LexA repressor, which in turn results in derepression of the SOS regulon. While most of the SOS genes are involved in DNA-repair, some carry out other functions, such as the inhibition of cell division. In this context, SulA (which is regulated by LexA) physically inhibits FtsZ polymerization and causes the formation of non-septated bacterial filaments, in order to prevent transmission of damaged DNA to daughter cells. In absence of SOS induction, however, direct chemical inhibition of FtsZ can also lead to bacterial elongation [8].

While reports describing conditions that induce *P. putida* filamentation are scarce, filamentation of other bacteria has been shown in response to DNA damage (as described above), nutrient deprivation, low temperature, media composition, low shaking speed and high osmolarity [6,9-11]. Additionally, the different stages of biofilm development in *P. putida* have been associated with alterations in bacterial length [12]. Furthermore, the plant-produced alkaloid berberine was found recently to induce filamentation in *Escherichia coli* K12 [8]. Collectively, these studies indicate that conditions and/or products encountered by *P. putida* during its natural life cycle could induce filamentation.

For a variety of (opportunistic) pathogens, the filamentous morphology has been shown to provide survival advantages [7]. More specifically, uropathogenic *Escherichia coli* (UPEC) filaments were more proficient in evading neutrophil phagocytosis compared to non-filamented UPEC [13]. UPEC filamentation was presumably induced in response to effectors of the host innate immunity. The intracellular survival of *Salmonella enterica* serovar Typhimurium in macrophages *in vitro* is also associated with a filamentous phenotype, which is probably induced by macrophage production of nitric oxide radicals [14]. In addition, filamentation has been shown to play a role in the infection process of, among others, *Proteus mirabilis*, *Legionella pneumophila*, *Mycobacterium tuberculosis* and *Shigella flexneri*[7]*.*

It remains unclear which mechanisms are at the origin of *P. putida* filamentation, which metabolic changes occur in *P. putida* filaments, and whether the *P. putida* filamented phenotype could confer environmentally advantageous traits. This study is the first to assess the global proteome and stress resistance of *P. putida* KT2440 when grown in conditions that induce filamentation.

## Results

### Morphologic and growth analysis of *P. putida* KT2440 grown in filament and non-filament inducing conditions

**Figure 1:**
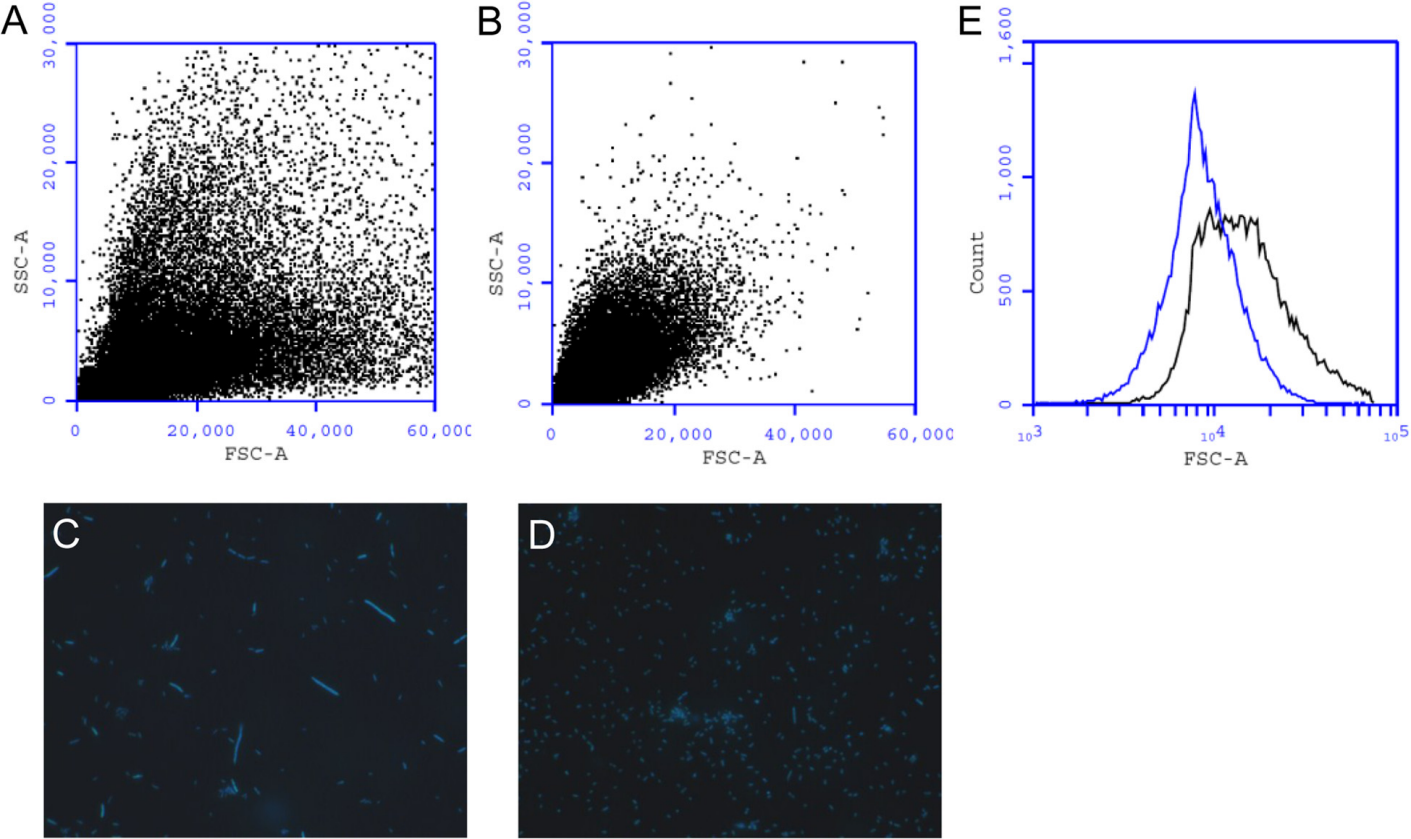
**Morphologic analysis of *P. putida *KT2440 grown at 50 and 150 rpm. **Flow cytometry dot plot (forward scatter versus side scatter) of *P. putida *KT2440 grown at 50 rpm (**A**) and 150 rpm (**B**). Microscopic imaging of Hoechst-stained *P. putida* KT2440 grown at 50 rpm (**C**) and 150 rpm (**D**) (magnification = 1000x). Flow cytometry histogram of *P. putida *grown at 50 rpm (black line) and 150 rpm (blue line) (**E**), representing the average bacterial length.

**Figure 2:**
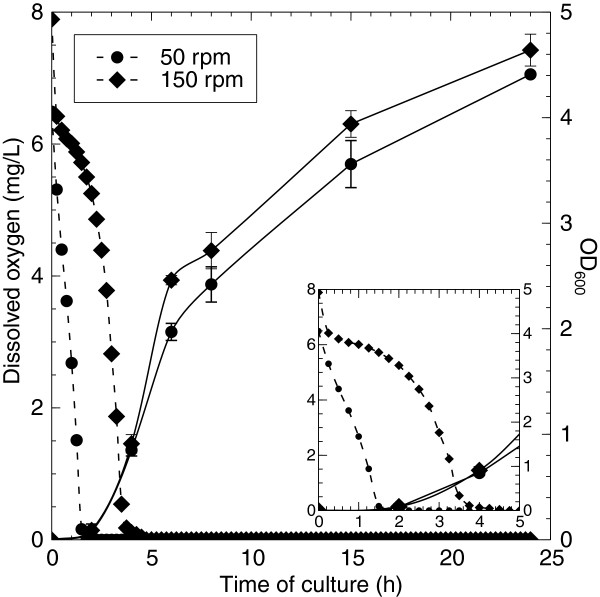
**Growth curves (black line) and dissolved oxygen concentrations (striped line) of 50 (circles) and 150 (diamonds) rpm cultures of *P. putida *KT2440 (inset showing zoom on first hours).**

The formation of filaments by *P. putida* KT2440 cultures was induced by overnight shaking at low speed (i.e., 50 rpm) [6], and corroborated by microscopic and flow cytometry analysis (Figure 1A and C). A bacterial culture shaken at high speed (i.e., 150 rpm) was used as a non-filamentous control (Figure 1B and D). Figure 1 demonstrates a clear difference in population heterogeneity between 50 rpm and 150 rpm-grown *P. putida* KT2440, with 50 rpm-grown bacteria showing an increased size distribution (based on forward scatter). The increase in bacterial size for 50 rpm-grown *P. putida* is also reflected in the comparative flow cytometry histogram (Figure 1E). Nucleic acid staining of 50 rpm and 150 rpm-grown bacteria (Figure 1C and D) confirmed the size differences. In order to rule out any effects of differences in growth phase between the two test conditions, the growth of *P. putida* KT2440 as a function of shaking speed was determined (Figure 2). No statistically significant (p<0.05) differences were found, only a slight significant increase in cell numbers was observed at 6 h for the 150 rpm-grown cultures. In agreement with the OD measurements, no statistically significant (p<0.05) differences were observed at 15 h in viable counts nor in biomass (45.3 ± 1.6 mg wet weight/5 mL for 50-rpm and 44.1 ± 0.9 mg weight/5 mL for 150-rpm cultures). As differences in the dissolved oxygen concentrations are expected to occur at different shaking speeds, the dissolved oxygen was measured for 50 rpm and 150 rpm-grown bacteria as a function of culture time. As presented in Figure 2, 50 rpm cultures reached undetectable oxygen levels after approximately 1.75 h, while this was only after 4 h for 150 rpm. Further, the maximum oxygen transfer rate at 150 rpm, calculated based on [15], was approximately 2.5 times higher than at 50 rpm.

### Stress resistance of *P. putida* KT2440 grown in filament and non-filament inducing conditions

**Figure 3:**
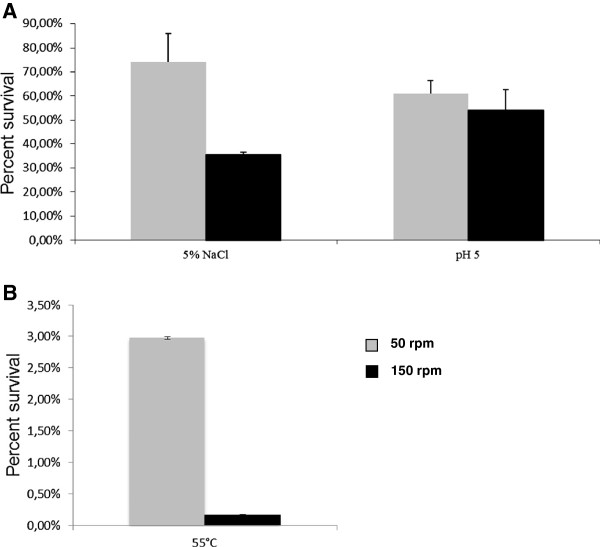
**Resistance profile of *P. putida *KT2440 exposed to 5% NaCl and 10**^**-4 **^**M citric acid (A), and 55°C (B) for 30 min following growth at 50 and 150 rpm.**

The stress resistance of *P. putida* KT2440 grown in filament-inducing and non-filament-inducing conditions (15 hours of growth) was investigated. *P. putida* KT2440 grown at 50 rpm demonstrated an increased resistance to heat shock (12.5-fold, p = 0.003) and saline stress (2.1-fold, p = 0.005), when compared to cells grown at 150 rpm (Figure 3). The acid stress resistance profile was similar for cultures grown at both tested shaking speeds.

### Proteomic analysis of *P. putida* KT2440 grown in filament and non-filament inducing conditions

**Figure 4:**
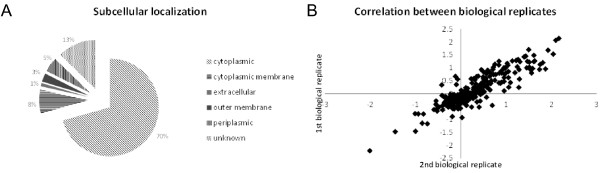
**Subcellular localization prediction using PSORTb revealed that identified proteins mainly belong to cytoplasmic compartment and cytoplasmic membrane (A). **Correlation between fold changes reached 0.89 which suggest high reproducibility of the proteomic data (**B**).

In order to investigate the molecular basis of the observed increased stress resistance of *P. putida* KT2440 grown in filament-inducing conditions, differential proteomic analysis was performed on samples after 15 hours of growth. This time point was chosen with the aim of obtaining an accumulation of effects associated with cultivating at different shaking speeds. Two biological replicates were analyzed, using a post-digest ICPL protocol, allowing the identification of 659 unique proteins, of which 542 were quantified. Subcellular localization prediction using PSORTb revealed that identified proteins mainly belonged to the cytoplasmic compartment and cytoplasmic membrane (Figure 4A). Almost 300 proteins could be quantified in both biological replicates and the calculated correlation between the 2 datasets reached 0.89, suggesting a very high reproducibility of our observations (Figure 4B). Finally, among the 542 quantified proteins, 223 proteins had a fold change lower than 0.66 or higher than 1.5 revealing that the difference in shaking speed had a major influence on the proteome of *P. putida* KT2440. The heat shock protein IbpA was induced the most in filament-inducing conditions (8.33 fold), followed by periplasmic phosphate-binding proteins (PP_2656, 4.26 fold; PP_5329, 3.33 fold). The RecA protein was induced 2.35 fold (Table 1). Among the differentially regulated proteins, a majority was involved in metabolic activity (Table 1). Altered metabolic activity in *P. putida* filaments was reflected in (i) down-regulation of a protein involved in purine/pyrimidine catabolism (PP_4038, 0.26-fold), (ii) down-regulation of proteins involved in the degradation of allantoate (PP_4034, 0.38-fold) and formation/downstream catabolism of urea (PP_0999, 0.23-fold; PP_1000, 0.28-fold; PP_1001, 0.24-fold) and glyoxylate (PP_4116, 0.27-fold; PP_2112, 0.42-fold and PP_4011, 0.25-fold), (iii) down-regulation of proteins involved in the production of ATP (PP_1478, 0.23-fold; PP_0126, 0.37-fold and PP_1478, 0.23-fold), (iv) differential expression of proteins involved in the metabolism of amino acids (PP_4666, 0.24-fold; PP_4667, 0.28-fold; PP_3433, 0.25-fold and PP_4490, 0.47-fold). In addition, proteomic analysis of *P. putida* filaments indicated down-regulation of formate metabolism (PP_0328, 0.38-fold), lipid degradation (PP_3282, 0.21-fold) and synthesis of polyhydroxyalkanoate (PP_5007, 0.33-fold).

**Table 1:** **Comparative proteome profile of *P. putida *grown at 50 rpm and 150 rpm**

**Locus tag**	**Protein name**	**Accession number**	**Fold-change**	**Protein function**
***Up-regulated proteins (50 rpm/150 rpm)***				
PP_0234	OprE	gi|26986977	2.41*	Outer membrane porin
PP_0268	OprQ	gi|26987010	1.80	Outer membrane porin
PP_0465	RplX	gi|26987206	1.61	50S ribosomal protein L24
PP_0812	CyoA	gi|26987548	1.82	Ubiquinol oxidase subunit 2
PP_0988	GcvP-1	gi|26987724	2.53	Glycine dehydrogenase
PP_1037	PurL	gi|26987773	1.59*	Phosphoribosylformylglycinamidine synthase
PP_1099		gi|26987835	1.74	Cold-shock domain-contain protein
PP_1629	RecA	gi|26988361	2.35*	Recombinase A
PP_1868		gi|26988598	2.25*	DEAD-box ATP dependent DNA helicase
PP_1982	IbpA	gi|26988708	8.33*	Heat shock protein Hsp20
PP_2468	RplT	gi|26989191	1.64	50S ribosomal protein L20
PP_2645	MgtB	gi|26989364	2.67*	Magnesium-translocating P-type ATPase
PP_2656	PstS	gi|26989375	4.26*	Phosphate ABC transporter, periplasmic phosphate-binding protein
PP_4718	FtsH	gi|26991401	2.04	ATP-dependent metalloprotease FtsH
PP_4803	DacA	gi|26991483	1.96*	Serine-type D-Ala-D-Ala carboxypeptidase
PP_5329	PstS	gi|26992005	3.33*	Phosphate ABC transporter phosphate-binding protein
PP_0460		gi|24981839	1.65	Ribosomal protein S3
***Down-regulated proteins (50 rpm/150 rpm)***				
PP_0126		gi|26986871	0.37*	Cytochrome c4
PP_0258		gi|26987000	0.21*	Hypothetical protein PP_0258
PP_0296		gi|26987038	0.36*	Glycine betaine/L-proline ABC transporter, periplasmic binding protein
PP_0308		gi|26987050	0.37	Membrane dipeptidase
PP_0315		gi|26987057	0.22	Rieske (2Fe-2S) domain protein
PP_0322	GlyA-1	gi|26987064	0.44	Serine hydroxymethyltransferase
PP_0328	FdhA	gi|26987070	0.38*	Formaldehyde dehydrogenase, glutathione-independent
PP_0382		gi|26987124	0.41	Nitrilase/cyanide hydratase and apolipoprotein N-acyltransferase
PP_0395		gi|26987137	0.19	Hypothetical protein PP_0395
PP_0397		gi|26987139	0.28*	Putative serine protein kinase, PrkA
PP_0541		gi|26987279	0.28	Acetyltransferase
PP_0545		gi|26987283	0.43*	Aldehyde dehydrogenase family protein
PP_0763		gi|26987499	0.50	Acyl-CoA synthetase
PP_0765		gi|26987501	0.45*	Hypothetical protein PP_0765
PP_0951	RpoX	gi|26987687	0.34*	Sigma 54 modulation protein/ribosomal protein S30EA
PP_0999	ArcC	gi|26987735	0.23*	Carbamate kinase
PP_1000	ArgI	gi|26987736	0.28*	Ornithine carbamoyltransferase
PP_1001	ArcA	gi|26987737	0.24*	Arginine deiminase
PP_1015		gi|26987751	0.52	Sugar ABC transporter, periplasmic sugar-binding protein
PP_1081		gi|26987817	0.44*	Glutaredoxin-related protein
PP_1084		gi|26987820	0.42	Anti-oxidant AhpCTSA family protein
PP_1122		gi|26987858	0.22	OmpA/MotB domain protein
PP_1210		gi|26987945	0.32*	DNA-binding stress protein, putative
PP_1478		gi|26988211	0.23*	NADH:flavin oxidoreductase/NADH oxidase
PP_1487		gi|26988220	0.40*	Hypothetical protein PP_1487
PP_1506	Adk	gi|26988238	0.34*	Adenylate kinase
PP_1661		gi|26988393	0.41*	Dehydrogenase subunit, putative
PP_1741		gi|26988472	0.28*	Substrate-binding region of ABC-type glycine betaine transport system
PP_1859	Ohr	gi|26988589	0.16*	OsmC family protein
PP_2006		gi|26988731	0.12*	Hypothetical protein PP_2006
PP_2105		gi|26988830	0.48	Hypothetical protein PP_2105
PP_2112	AcnA	gi|26988836	0.42*	Aconitate hydratase
PP_2140		gi|26988864	0.47	Hypothetical protein PP_2140
PP_2303	HupB	gi|26989027	0.52	Histone family protein DNA-binding protein
PP_3089		gi|26989808	0.37*	Hypothetical protein PP_3089
PP_3232		gi|26989950	0.16*	Acetyltransferase
PP_3283	PhaB	gi|26990001	0.21*	Enoyl-CoA hydratase
PP_3433	Hpd	gi|26990146	0.25*	4-hydroxyphenylpyruvate dioxygenase
PP_3611		gi|26990322	0.12*	Hypothetical protein PP_3611
PP_3668		gi|26990379	0.28*	Catalase/peroxidase HPI
PP_3765		gi|26990470	0.24*	Transcriptional regulator MvaT, P16 subunit, putative
PP_3839	AdhA	gi|26990544	0.30*	Alcohol dehydrogenase
PP_4011	Icd	gi|26990716	0.25*	Isocitrate dehydrogenase, NADP-dependent
PP_4034		gi|26990737	0.38*	Allantoate amidohydrolase
PP_4037		gi|26990739	0.32*	Putative oxidoreductase
PP_4038		gi|26990740	0.26*	Dihydropyrimidine dehydrogenase
PP_4116	AceA	gi|26990810	0.27*	Isocitrate lyase
PP_4486		gi|26991172	0.51	Cationic amino acid ABC transporter, periplasmic binding protein
PP_4490	PhhA	gi|26991176	0.47*	Phenylalanine 4-monooxygenase
PP_4593		gi|26991277	0.20*	Hypothetical protein PP_4593
PP_4666	MmsB	gi|26991350	0.24*	3-hydroxyisobutyrate dehydrogenase
PP_4667	MmsA-2	gi|26991351	0.28*	Methylmalonate-semialdehyde dehydrogenase
PP_4848		gi|26991528	0.54	DnaJ family curved-DNA-binding protein
PP_4870		gi|26991550	0.38*	Azurin
PP_5007		gi|26991684	0.33*	Poly(hydroxyalkanoate) granule-associated protein
PP_5220	ElbB	gi|26991896	0.45	Isoprenoid biosynthesis protein
PP_5232		gi|26991908	0.48	Hypothetical protein PP_5232
PP_5258		gi|26991934	0.27*	Aldehyde dehydrogenase family protein
PP_5260		gi|26991936	0.24*	Hypothetical protein PP_5260

* P-value < 0.05.

### Role of RecA in *P. putida* KT2440 filamentation and stress resistance

The increased abundance of RecA (PP_1629, 2.35-fold) in 50 rpm cultures of *P. putida* KT2440 (Table 1) suggested the activation of the SOS response. Since only induction of RecA was observed, this could indicate a mild SOS response [16]. In addition, the heterogeneity of the SOS response at single cell level could be masked at the population level [17]. This heterogeneity was also apparent in cell morphology between 50 rpm- and 150 rpm-grown *P. putida* KT2440 (Figure 1). In order to determine whether 50 rpm-induced filamentation in *P. putida* KT2440 was indeed dependent on RecA, an isogenic *recA* mutant cultured in 50 and 150 rpm conditions was examined. Intriguingly, the 50 rpm-grown *P. putida* KT2440 *recA* mutant filamented at similar levels as the wild type *P. putida* KT2440 (Additional file 1: Figure S1). In contrast to filamentation, the increased heat shock resistance of *P. putida* KT2440 grown at 50 rpm was RecA-dependent, since an isogenic *recA* mutant was equally resistant to heat shock when grown at 50 rpm or 150 rpm (Additional file 2: Figure S2).

## Discussion

As a soil organism, *P. putida* recurrently encounters filament-inducing conditions during its natural life cycle. Our data indicate that filament formation of *P. putida* could confer environmentally advantageous traits. Indeed, *P. putida* KT2440 grown at low shaking speed produced filaments and was more resistant to heat shock and saline stress. Similar observations were made for *Caulobacter crescentus* filaments, which showed a higher resistance to oxidative, osmotic, thermal and acid stress [18].

The comparative proteome profile indicated that the metabolic activity of *P. putida* KT2440 grown at 50 rpm was significantly different from *P. putida* KT2440 grown at 150 rpm. The most pronounced induction occurred for the heat shock protein IbpA. This small heat shock protein belongs to the widely conserved family of α-crystallin-type heat shock proteins. The latter appears to play a very versatile role in the protection against different stress conditions via protein and membrane protection [19]. In addition, many small heat shock proteins form oligomers, which may vary by the degree of phosphorylation or ion concentration [20] (induction of PP_2645, PP_2656 and PP_5329).

Although no observable differences in dissolved oxygen levels could be reported at the time of proteomic analysis (i.e., 15 hours, below detection limit for both conditions) (Figure 2), this does not completely rule out the role of dissolved oxygen in the observed results as the maximum oxygen transfer rate at 150 rpm is approximately 2.5 times higher than at 50 rpm [15]. Ohr, a protein of the OsmC family (osmotically inducible protein) was 6.25-fold down-regulated in filamented *P. putida*, and is involved in the resistance to oxidative stressors, such as organic peroxide, but not in osmotic stress resistance [21]. In addition to a decreased Ohr abundance, other proteins involved in oxidative stress resistance were present at lower levels in 50 rpm samples, including a catalase/peroxidase (PP_3668, 0.28-fold), an antioxidant AhpC (PP_1084, 0.42-fold), a glutaredoxin-related protein (PP_1081, 0.44 fold) and a putative DNA binding stress protein (PP_1210, 0.32-fold). The latter has recently been described as an oxidative stress-inducible Dps miniferritin [22,23], and was found up-regulated in an OxyR mutant of *P. aeruginosa*[23]. The differential abundance of proteins involved in oxidative stress resistance could potentially be explained by lower oxygen levels in 50 rpm cultures (and/or decreased catabolism). The increase of OprE (PP_0234, 2.41-fold) and CyoA (PP_0812, 1.82-fold) further suggests limitations in oxygen availability in 50 rpm cultures [24,25]. Finally, oxygen limitation is related to bacterial filamentation and/or RecA induction [6,26-28]. However, Jenssen and colleagues determined that, in addition to oxygen deprivation, additional factors such as exhaustion of medium components and changes in growth rate, were important for *P. putida* filamentation [6].

While RecA was more abundant in *P. putida* KT2440 grown at 50 rpm, the *P. putida* KT2440 *recA* mutant filamented at similar levels as the wild type. A similar observation was reported previously, showing that an *E. coli recA* mutant displayed similar levels of filamentation as the wild type strain in response to growth at high pressure, despite strong evidence of RecA-mediated SOS response activation [29-31]. Gottesman *et al*. (1981) suggested the existence of a transient filamentation phenotype in response to UV, independent of SulA [32], which could explain the RecA-independent filamentation phenotype of 50 rpm-grown *P. putida* KT2440 in the present study.

While the bacterial SOS response and associated filamentation is typically triggered by treatments directly affecting DNA integrity (e.g. exposure to mitomycin C or UV), a number of environmental conditions were reported to cause DNA damage in an indirect manner (e.g. starvation, aging, β-lactam antibiotics and high pressure stress) [30,33-36]. As such, high pressure-induced filamentation of *E. coli* was shown to stem from the activation of a cryptic Type IV restriction endonuclease (i.e. Mrr) endogenously present in the cell [37], while β-lactam antibiotics triggered DpiA to interfere with DNA replication [30,36]. Even though it remains unclear which metabolic changes could indirectly lead to DNA damage and SOS response activation, the major changes in metabolism provide evidence for new triggers of the SOS response.

## Conclusion

In conclusion, our data indicate that filament-formation of *P. putida* KT2440 could confer environmentally advantageous traits, by increasing its resistance to saline and heat shock. We demonstrated that culturing at low shaking speed induced expression of RecA, which plays a central role in the SOS response, putatively through changes in amino acid metabolism and/or oxygen availability. Furthermore, the increased heat shock resistance was found to be RecA dependent. Filamentation could thus represent an adaptive survival strategy of *P. putida*, allowing it to persist during times of elevated soil temperatures, increased osmolarity (e.g., due to soil water evaporation) and/or increased pollution.

## Methods

### Bacterial strains, media and growth conditions

*P. putida* KT2440 (ATCC 12633) and its isogenic *recA* mutant derivative (kindly provided by Juan-Luis Ramos) were used in the present study. The bacterial strains were grown in Luria Bertani (LB) medium at 30°C. For incubation at different shaking speeds, an overnight shaking culture (150 rpm) of *P. putida* was diluted 100x in fresh LB medium. Ten milliliters of the dilution were transferred into 50 ml Erlenmeyer flasks. The flasks were placed on an orbital shaker at 50 rpm (filament-inducing condition) or at 150 rpm (non-filament-inducing condition) [6]. Growth was monitored by measuring optical density at the 600 nm (OD_600_).

### Analysis of dissolved oxygen levels

The measurement of the dissolved oxygen (DO) concentration of 50 and 150 rpm cultures was performed using a Knick KNI913 oxygen meter. DO levels were measured during culture, at 15 min intervals for 24 hours.

### Environmental stress assays

The assessment of cell viability following exposure to saline, acid and thermal stress was performed on *P. putida* KT2440 grown at 50 and 150 rpm for 15 hours as described previously [38]. The concentrations of each stress agent were as follows: 5% NaCl for osmotic stress and 10^-4^ M citric acid for acid stress resistance (pH = 5). For heat shock, exposure of cultures to a temperature of 55°C was applied. Cells were exposed to each stress for 30 minutes. Bacteria were diluted and plated on LB agar before and after exposure to the stress factors in order to determine the survival percentage.

### Bacterial morphology

The morphology of *P. putida* KT2440 following incubation at different shaking speeds was visualized by fluorescence microscopy of Hoechst stained cells. Briefly, 600 μl of bacterial culture (after 15 hours of growth) was resuspended in 500 μl 70% ethanol to fix the cells, incubated at room temperature for 20 min and resuspended in saline solution. Next, 2.5 μl Hoechst solution (200 μg/ml) (Hoechst 33258, Sigma-Aldrich, Belgium) was added and incubated for 20 min. Five microliters of this suspension was transferred to a microscopic glass slide, covered with a coverslip and analyzed with a Zeiss Axiovert 100M fluorescence microscope (350 nm filter, 100x oil objective). Acquisition of images was performed with an Axiocam and further processed using the Axiovision software package.

### Flow cytometry analysis

*P. putida* KT2440 grown at different shaking speeds was analyzed with an Accuri C6 flow cytometer (Accuri Cytometers) to assess the average cell length. Forward and side scatter signals were measured and a total of at least 10,000 cells were recorded for each sample. The respective cell populations were delimited to eliminate background signals originating from cell debris. All data analysis was performed with the CFlow Software.

### Proteomics

Protein extraction and analysis was performed on *P. putida* grown at 50 and 150 rpm for 15 hours. Proteins were extracted and labeled isotopically using ICPL, and the post-digest procedure was performed as described in [39]. Labeled tryptic peptides were submitted to online 2D-LC separation prior to MS/MS analysis as described previously [39], except that SCX column was eluted with 11 plugs of increasing NH_4_Cl concentration (5, 10, 25, 50, 75, 100, 125, 150, 200, 400 and 800 mM in loading solvent).

For MS/MS data processing, peptide peaks were detected and processed using Mascot Distiller (version 2.3.2). Created peak list was used as the input for Mascot MS/MS Ions searches using an in-house Mascot 2.2 server (Matrix Science) against the NCBInr database restricted to *Pseudomonas putida* (KT2440). The search parameters used were: enzyme = trypsin; Max. Missed cleavages = 2; Fixed modifications = Carbamidomethyl (C); Variable modifications = Oxidation (M); ICPL modification at both peptide N-ter and lysine side chain. Peptide tolerance ± 1.3 Da; MS/MS tolerance ± 0.5 Da; Peptide charge = 2+ and 3+; Instrument = ESI-TRAP. Only proteins identified with a protein score above the calculated Mascot ion score, defined as the 95% confidence level, were considered.

Mascot distiller was also used for protein quantification with parameters as follows: integration method: simple; correlation threshold: 0.8; standard error threshold: 999; Xic threshold: 0.2; max Xic width: 7; fraction threshold: 0.5 and mass time matches allowed. Only peptides with an ion score above 30 were considered for quantification. The protein ratio corresponds to the average of peptide ratios. After examination that the distribution of protein ratios was almost centered on 1, a normalization based on the median of the peptide ratios was realized by mascot distiller on the complete dataset. Proteins with fold changes above 1.5 or below 0.66 were considered as in modified abundance.

### Statistical analysis

All experiments were performed in triplicate, unless stated otherwise. The statistical determination of significance (α = 0.05) was calculated using a Student’s *t*-test on the biological replicates of each experimental condition.

## Competing interest

The authors declare that no competing interests exist.

## Authors’ contributions

AC and RVH designed the study; contributed to the acquisition, analysis and interpretation of data, and wrote the manuscript. BL and RW performed proteomic analysis and data interpretation. AA assisted in data interpretation and contributed to manuscript writing. PC contributed to data interpretation, and NL helped to draft the manuscript. All authors read and approved the final manuscript.

## Supplementary Material

Additional file 1 **Figure S1. **Morphologic analysis of a *P. putida *KT2440 isogenic *recA *mutant grown at 50 rpm and 150 rpm. Flow cytometry dot plot (forward scatter versus side scatter) of *P. putida* KT2440 *recA *mutant grown at 50 rpm (A) and 150 rpm (B). Microscopic imaging of Hoechst-stained *P. putida* KT2440 *recA *mutant grown at 50 rpm (C) and 150 rpm (D) (magnification = 1000x). Flow cytometry histogram of *P. putida *KT2440 *recA *mutant grown at 50 rpm (grey line) and 150 rpm (black line) (E), representing the average bacterial length. Click here for file

Additional file 2 **Figure S2. **3 Heat shock resistance of a *P. putida *KT2440 isogenic *recA* mutant grown at 50 and 150 rpm, as compared to wild type. Bacteria were exposed to 55°C during 30 min. Click here for file
