# Supplementary figures and images for: Differential proteomics and physiology of Pseudomonas putida KT2440 under filament-inducing conditions
Source: BMC Microbiol. 2012 Nov 27;12:282. doi: 10.1186/1471-2180-12-282 (PMC3538555; doi:10.1186/1471-2180-12-282)

## Slide 1
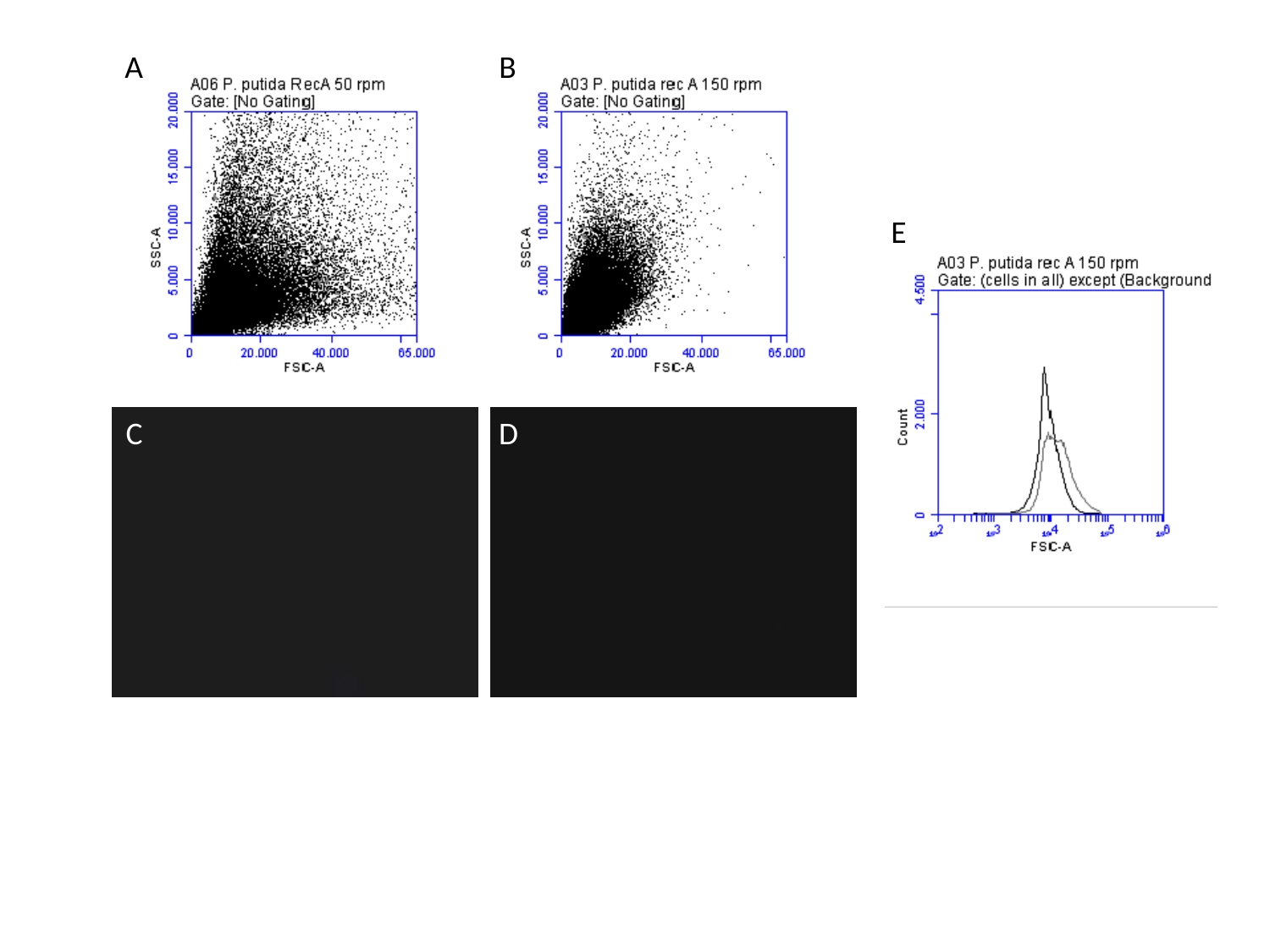

A
B
E
C
D

Supplement: Additional file 1 — Figure S1. Morphologic analysis of a P. putida KT2440 isogenic recA mutant grown at 50 rpm and 150 rpm. Flow cytometry dot plot (forward scatter versus side scatter) of P. putida KT2440 recA mutant grown at 50 rpm (A) and 150 rpm (B). Microscopic imaging of Hoechst-stained P. putida KT2440 recA mutant grown at 50 rpm (C) and 150 rpm (D) (magnification = 1000x). Flow cytometry histogram of P. putida KT2440 recA mutant grown at 50 rpm (grey line) and 150 rpm (black line) (E), representing the average bacterial length. [file 1471-2180-12-282-S1.ppt]

## Slide 1
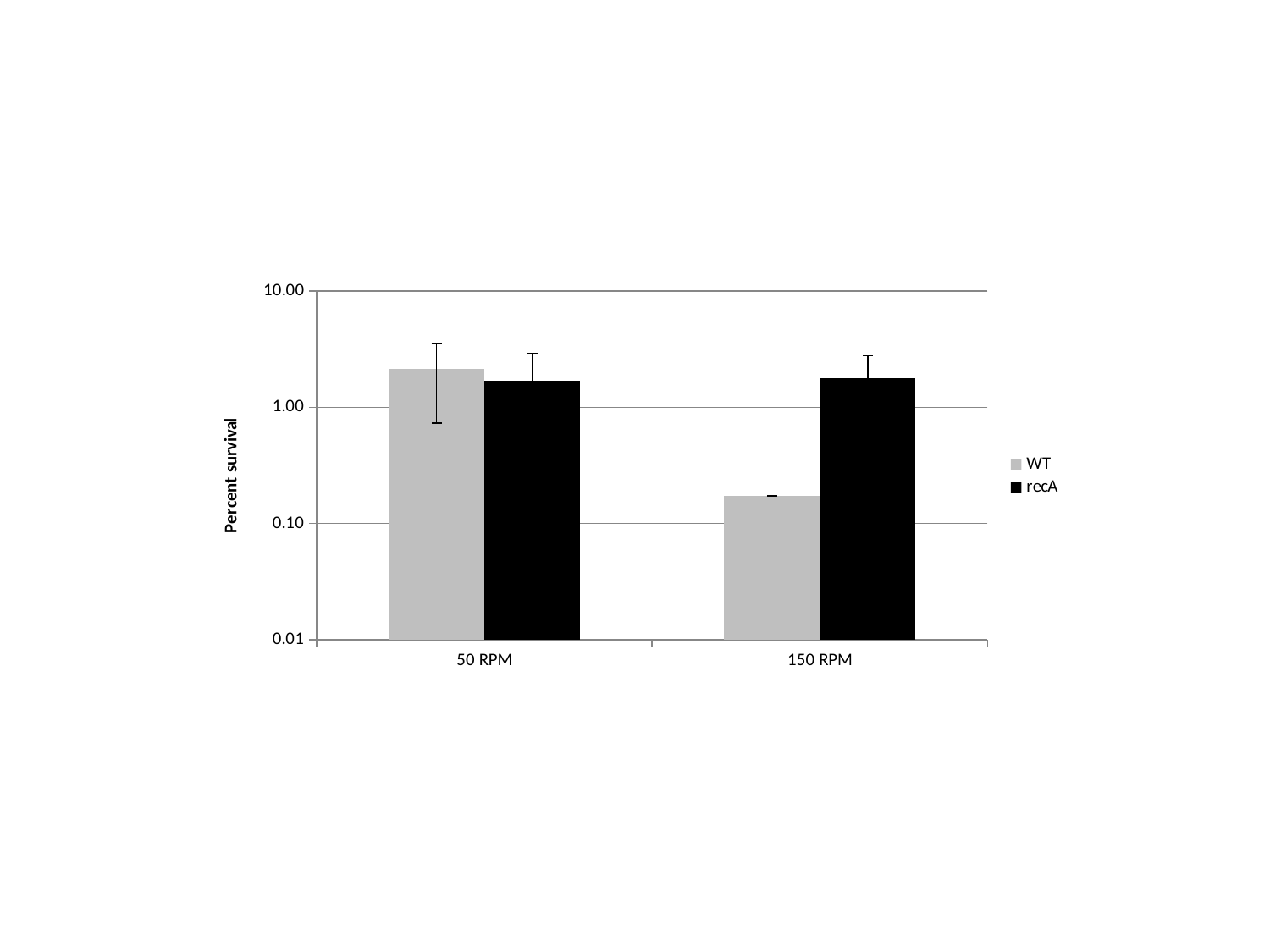

### Chart
| Category | | |
|---|---|---|
| 50 RPM | 2.147334214027943 | 1.687108490814339 |
| 150 RPM | 0.17167698202181 | 1.763975966562173 |

Supplement: Additional file 2 — Figure S2. 3 Heat shock resistance of a P. putida KT2440 isogenic recA mutant grown at 50 and 150 rpm, as compared to wild type. Bacteria were exposed to 55°C during 30 min. [file 1471-2180-12-282-S2.pptx]
